# Validation of the BATT score for prehospital risk stratification of traumatic haemorrhagic death: usefulness for tranexamic acid treatment criteria

**DOI:** 10.1186/s13049-020-00827-5

**Published:** 2021-01-06

**Authors:** Francois-Xavier Ageron, Timothy J. Coats, Vincent Darioli, Ian Roberts

**Affiliations:** 1 Clinical Trials Unit, London School of Hygiene & Tropical Medicine, London, WC1E 7HT UK; 2 Department of Emergency Medicine, Lausanne University Hospital, University of Lausanne, 1011 Lausanne, Switzerland; 3 Emergency Medicine, University of Leicester, Leicester, UK

**Keywords:** Trauma, Tranexamic acid, Bleeding, Score, Prognostic model

## Abstract

**Background:**

Tranexamic acid reduces surgical blood loss and reduces deaths from bleeding in trauma patients. Tranexamic acid must be given urgently, preferably by paramedics at the scene of the injury or in the ambulance. We developed a simple score (Bleeding Audit Triage Trauma score) to predict death from bleeding.

**Methods:**

We conducted an external validation of the BATT score using data from the UK Trauma Audit Research Network (TARN) from 1st January 2017 to 31st December 2018. We evaluated the impact of tranexamic acid treatment thresholds in trauma patients.

**Results:**

We included 104,862 trauma patients with an injury severity score of 9 or above. Tranexamic acid was administered to 9915 (9%) patients. Of these 5185 (52%) received prehospital tranexamic acid. The BATT score had good accuracy (Brier score = 6%) and good discrimination (C-statistic 0.90; 95% CI 0.89–0.91). Calibration in the large showed no substantial difference between predicted and observed death due to bleeding (1.15% versus 1.16%, *P* = 0.81). Pre-hospital tranexamic acid treatment of trauma patients with a BATT score of 2 or more would avoid 210 bleeding deaths by treating 61,598 patients instead of avoiding 55 deaths by treating 9915 as currently.

**Conclusion:**

The BATT score identifies trauma patient at risk of significant haemorrhage. A score of 2 or more would be an appropriate threshold for pre-hospital tranexamic acid treatment.

**Supplementary Information:**

The online version contains supplementary material available at 10.1186/s13049-020-00827-5.

## Introduction

Tranexamic acid (TXA) reduces surgical blood loss and reduces deaths from bleeding in trauma patients [1, 2]. TXA must be given urgently, preferably by paramedics at the scene of the injury or in the ambulance [3]. Many bleeding deaths occur soon after injury and there is a 10% reduction in treatment effectiveness for every 15 min treatment delay [4]. Paramedics need clear criteria that can be applied at the scene to guide who to treat. We previously developed a prognostic model to predict death from bleeding and showed that the relative reduction in mortality with TXA does not vary with baseline risk [5, 6]. Because many deaths are in patients at low and intermediate risk, TXA use should not be restricted to the most severely injured [6]. In this study, we derive a simple score that paramedics can use at the scene to help decide who to treat with TXA. We conduct an external validation of the score and explore different TXA treatment thresholds.

## Method

We developed a simple score (Bleeding Audit and Triage Trauma Score - BATT) to predict death due to bleeding in trauma patients. We conducted an external validation of this score using data from the UK Trauma Audit Research Network (TARN) from 1st January 2017 to 31st December 2018. Finally, we evaluated the impact of TXA treatment thresholds in trauma patients.

### Development of the BATT score

We previously developed and validated a prognostic model to predict death due to bleeding in trauma patients. The methods are described in detail elsewhere [5]. Briefly, data on bleeding trauma patients from 298 hospitals in 41 countries were used to derive the model. We validated the model using an internal–external cross-validation method based on data from 41 countries to ensure that the results are widely applicable. The final prognostic model included age, systolic blood pressure, Glasgow Coma Scale, heart rate, respiratory rate and mechanism of injury. To develop the BATT score, we assigned points for each predictor that were proportional to the coefficients of the regression equation. We added the criterion high velocity trauma as the intercept of the regression equation corresponding to the inclusion criteria of the trauma registry used for the development of prognostic model. High velocity trauma is routinely assessed at the scene and corresponds to injury from road traffic crash (with intrusion, ejection, death in same passenger compartment, and motor vehicle versus pedestrian or bicyclist), fall from high height (> 3 m), blow or blast [7]. An electronic version of the score is available for computer or smartphone: https://www.evidencio.com/models/show/1393

### Validation of the BATT score

We used data from the Trauma Audit Research Network (TARN) from 1st January 2017 to 31st December 2018 to validate the BATT score for use in England and Wales. The TARN database includes data on patients with an Injury Severity Score (ISS) of nine or more who are admitted to hospital in England and Wales for at least three nights, died in hospital or were transferred to another hospital for specialist care [8]. The exclusion criteria were isolated mild traumatic brain injury with loss of consciousness, superficial scalp injury, patients 65 years or older with femoral neck or single pubic rami fracture, fracture or dislocation of the foot or hand, closed fracture or dislocation of an isolated limb, simple skin laceration with blood loss < 20%.

Because death due to bleeding is not recorded in the TARN database, we used early deaths and early deaths with evidence of haemorrhage as a proxy for death due to bleeding. Causes of trauma deaths depend on time and location of death [9]. Prehospital immediate deaths are likely to be due to traumatic brain injury or cardiovascular injuries [10]. The main causes of in-hospital deaths are exsanguination and brain injury [11]. Two studies, one in North America and one including two large European registries (UK and Germany) showed that deaths due to exsanguination occurred within 24 h with a peak at 6 h after admission [9, 12]. Deaths due to head injuries occurred within 72 h with a peak at 24 h after admission. Consequently, we included deaths from all cause within 12 h of injury (excluding asphyxia, drowning, hanging, or massive destruction of skull or brain) and deaths between 12 to 24 h with evidence of bleeding (activation of massive transfusion protocol or blood within 6 h or an abbreviated injury scale (AIS) diagnosis associated with haemorrhage listed in the Supplementary file 1).

We assessed the accuracy, discrimination and calibration of the BATT score. Accuracy was assessed using the Brier score. Because the Brier score depends on the prevalence of the outcome, we also calculated the scaled Brier score to account for the baseline risk of death due to bleeding (Supplementary file 2). The scaled Brier score ranges from 0 to 100% and indicates the degree of error in prediction [13]. A scaled Brier score of 0% shows perfect accuracy. Discrimination is the ability of the score to correctly identify patients with the outcome. We estimated the sensitivity, specificity, positive and negative likelihood ratio for each threshold of the BATT score. The likelihood ratio is the likelihood of a positive score in a patient with the outcome compared to the likelihood of a positive score in a patient without the outcome [14]. The positive likelihood ratio is the ratio of sensitivity to 1-specificity. The negative likelihood ratio is the ratio of 1-sensitivity to specificity. A positive likelihood ratio of 10 or above will result in a large increase in the probability of the outcome. A negative likelihood ratio of 0.1 or less will result in a large decrease in the probability of the outcome. We plotted the Receiving Operating Characteristic (ROC) curve which is the sensitivity (true positives) on 1-specificity (false positives) for different threshold of the BATT score [15]. An ideal score will reach the upper left corner (all true positive with no false positive). We estimated the area under the ROC curve (AUROC) that corresponds to the concordance statistic (C-Statistic) for binary outcome. A C-statistic of 1.0 shows perfect discrimination ability. Calibration is the agreement between observed and predicted outcomes. We estimated calibration in the large as the difference between the mean predicted and observed probabilities and the ratio of the predicted and observed number of events (P/O). We also plotted the observed and predicted probabilities of death by decile of the score and with local regression based on LOESS algorithm [13]. We estimated the calibration intercept and slope of the calibration plot as a measure of spread between predicted and observed outcome. Ideally, the intercept would be zero indicating that the predictions are neither systematically too low or too high and the slope would be 1 [16]. There were missing value for some predictors but no missing outcome data. To estimate baseline risk for the full dataset, we replaced missing predictors using multiple imputation by chained equations on early death, age, systolic blood pressure, respiratory rate, heart rate, Glasgow coma scale, time for injury, time for prehospital ambulance arrival, and time for hospital admission with 20 imputed datasets.

### Evaluation of TXA treatment criteria

We evaluated two different TXA treatment strategies: (1) prehospital treatment of all trauma patients with an ISS ≥9 at the scene of the injury, (2) hospital treatment of all trauma patients with an ISS > 9 in the emergency department (ED). We compared each treatment strategy according to different thresholds of the BATT score to assess its clinical usefulness and treatment criteria.

We estimated the impact of TXA treatment for each treatment criteria. Since randomized trials of TXA in trauma patients report no increase in deaths from adverse events, the net impact of TXA was given by the number of deaths due to bleeding avoided by the treatment [6, 17]. To estimate the number of deaths avoided by TXA, we predicted the baseline risk of death due to bleeding using our previously published prognostic model [5]. To estimate post-treatment probabilities, we applied the treatment effect to these baseline risks taking into account time to treatment [4]. The risk difference was used to estimate the number of deaths avoided. To account for miscalibration of predicted baseline risks, we conducted a sensitivity analysis using observed early deaths with evidence of haemorrhage as baseline risks. The details of both modelling methods and equations are described in the Supplementary file 3. We plotted the cumulative number of death due to bleeding avoided by BATT score threshold in a decision curve analysis as described by Vickers et al. [18] We compared decision curve analysis for each scenario. We estimated the number needed to treat to save one life for each BATT score threshold and each scenario. The registry-based study design predetermines the sample size. All analyses were performed using STATA software (version 16.0; Stata Corp, College Station, TX, USA).

## Results

Table 1 shows the BATT Score. The minimum score is 0 and the maximum score is 27.

**Table 1:** BATT score

**Age**	≥ 65 years old	+ 1
	≥ 75 years old	+ 2
**Systolic Blood Pressure**	< 60 mmHg	+ 14
	≥ 60 and < 100 mmHg	+ 5
**Glasgow Coma Scale**	≤ 8	+ 4
	> 8 and ≤ 12	+ 3
**Respiratory rate**	< 10 or ≥ 30/min	+ 2
	Alt: Oxygen saturation < 90	+ 2
**Heart rate**	> 100/min	+ 1
**Penetrating injury**	Yes	+ 2
**High velocity trauma**	Yes	+ 2

The score is not suitable for isolated limb trauma or isolated neck femoral fracture in people older than 65 years

### External validation - patient’s characteristics

**Fig. 1:**
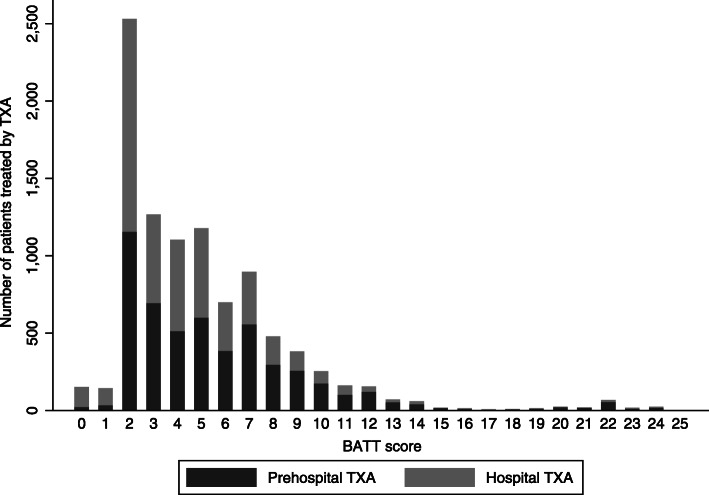
Number of patients treated with tranexamic acid by BATT score in UK TARN data

We validated the score in 104,862 trauma patients with an ISS ≥ 9 who were transported to hospital by ambulance in England and Wales between 2017 and 2018. Their characteristics are summarized in Table 2. The mean age was 62 years and 3189 (3%) had penetrating injuries. The median time from injury to ambulance arrival was 69 min, IQR (24–174). Mean ISS was 16 (± 9) and 46% of patients had an ISS ≥ 16. TXA was administered in 9915 (9%) patients. Of these 5185 (52%) received it prehospital. The median time from injury to treatment was 48 min, IQR (35–68) when TXA was given prehospital and 148 min, IQR (103–251) when it was given in hospital. 2760 (3%) of the trauma patients received TXA within 1 h and 5727 (6%) received TXA within 3 h of injury. The mean ISS of patients treated with TXA was 23 (±13) compared with 14 (±7) for patients who were not treated (*P* < 0.001). Most patients treated with TXA had a low or intermediate risk of death due to bleeding (Fig. 1). Most patients treated had a BATT score of 2. The proportion of patients who received prehospital TXA increased with the BATT score. There was no loss to follow-up at 30 days. A total of 2517 (2.4%) patients died within 24 h and 8874 (8.5%) died within 30 days. Early death with evidence of haemorrhage was reported for 1219 (1.2%) patients.

**Table 2:** Characteristics of the trauma patients used to validate the BATT score

	*N* = 104,862	Missing
Mean age (SD)	62 (24)	0
< 18, N (%)	5616 (5)	–
18–44, N (%)	19,744 (19)	–
45–64, N (%)	26,354 (25)	–
65–74, N (%)	13,123 (13)	–
≥75, N (%)	40,025 (38)	–
Sex female, N (%)	47,346 (45)	0
Penetrating injury, N (%)	3189 (3)	0
Circumstances, N (%)		0
Motor vehicle crash	19,709 (19)	–
Fall less than 2 m	65,573 (62)	–
Fall more than 2 m	10,604 (10)	–
Blast – Blow – Crush	5266 (5)	–
Shooting	234 (0)	–
Stabbing	2538 (2)	–
Other	1938 (2)	–
First systolic blood pressure, mean (SD)	138 (28)	12,450 (12)
First systolic blood pressure < 90 mmHg, N (%)	3033 (3)	
First Glasgow coma scale, N (%)		12,695 (12)
14–15	90,579 (86)	–
9–13	8566 (8)	–
3–8	5717 (6)	–
First heart rate, mean (SD)	86 (20)	11,479 (11)
Heart rate > 120 bpm, N (%)	5475 (5)	
Time from injury to ambulance arrival < 3 h, N (%)	79,430 (76)	50,496 (48)
Time from injury to hospital admission < 3 h, N (%)	63,246 (60)	50,465 (48)
Injury Severity Score, mean (SD)	16 (9)	0
ISS 9–15, N (%)	58,695 (56)	–
ISS 16–24, N (%)	24,635 (23)	–
ISS 25–34, N (%)	17,682 (17)	–
ISS ≥ 35, N (%)	3850 (4)	–
Tranexamic acid treatment	9915 (9)	13,115 (13)
Prehospital	5185 (5)	–
Hospital	4576 (4)	–
Unknown	176 (0.1)	
Any blood product received	4922 (5)	0
Massive transfusion protocol activated	2487 (2)	–
Blood received within 6 h of injury	2277 (2)	–

### External validation

The Table 3 shows the performance of the BATT score. The scaled Brier score was 6%. The receiving operator curve, the sensitivity and specificity at different thresholds of the BATT score are shown in Supplementary files 4 and 5. A threshold of 2 or more had a sensitivity of 99% and a negative likelihood ratio of 0.03. The C-statistic was 0.90; 95% CI (0.89–0.91). The observed (1.16%) and predicted (1.15%) probabilities of death due to bleeding were similar (*p* = 0.81). The calibration curve showed slight over-prediction in low risk patients and under-prediction in intermediate and high-risk patients (Supplementary file 6). The calibration intercept was close to zero (0.00032) with a calibration slope of 1.09 (Table 3).

**Table 3:** Performance of the BATT score

	BATT score	95% CI
Overall performance		
Brier score	0.0107	
Scaled Brier score (%)	6	
Discrimination		
C-statistic	0.90	0.89–0.91
Mean predicted death due to bleeding (%)		
If patient died from bleeding	6.5	
If patient did not die from bleeding	1.1	1.1–1.1
Discrimination slope (%)	5.4	0.053–0.056
Calibration		
Observed deaths due to bleeding (%)	1.16	1.1–1.2
Predicted deaths due to bleeding (%)	1.15	1.1–1.2
Calibration-in-the-large (%)	0.01	0.00–0.01
Ratio Predicted/Observed	0.99	0.94–1.05
Calibration Intercept	0.00032	
Calibration slope	1.09	1.07–1.11

### Clinical usefulness

**Fig. 2:**
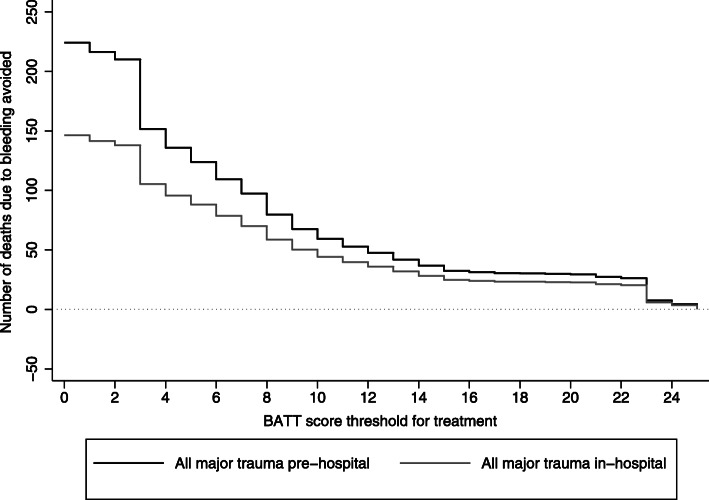
Impact of tranexamic acid treatment by BATT score threshold

**Fig. 3:**
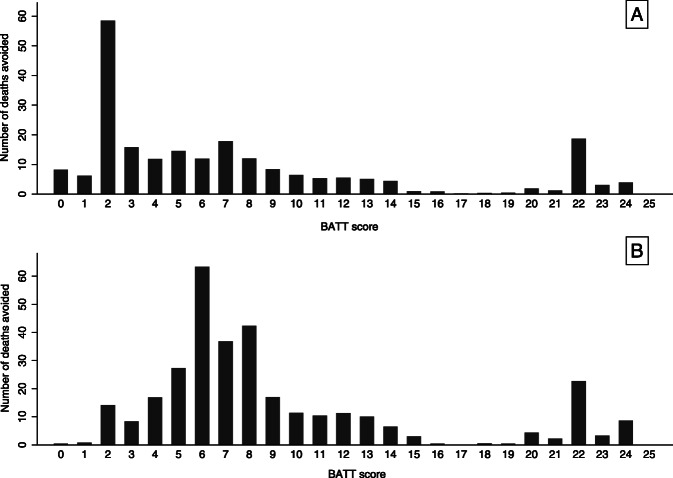
Number of deaths avoided due to prehospital tranexamic acid by BATT score. **a**: Estimated number of deaths avoided based on predicted baseline risk. **b**: Estimated number of deaths avoided based on observed probabilities of death (Sensitivity analysis)

Figure 2 is a decision curve analysis showing the number of deaths due to bleeding avoided by TXA treatment by BATT score threshold. Treating all trauma patients as soon as possible at scene or in the ambulance prevented more deaths than in hospital treatment. The cumulative number of deaths avoided decreased as the BATT score threshold increased. Table 4 shows the number of deaths avoided for the different scenarios and the sensitivity analysis based on observed early deaths in 2017 and 2018 in England and Wales. The sensitivity analysis confirms that prehospital treatment provides the maximum benefit with a lower number needed to treat than hospital treatment. Table 5 shows the number of deaths avoided and the number needed to treat for each BATT score threshold when patients are treated as soon as possible in the prehospital setting and within 3 h of injury. A BATT score treatment threshold of 2 corresponds to the treatment of 61,598 patients (59% of major trauma patients included in TARN registry with ISS ≥ 9) and results in 210 deaths avoided (Table 5). A BATT score treatment threshold below 2 resulted in 6 to 14 additional deaths avoided with an additional number needed to treat for one death avoided more than 1000 patients (Table 5, Fig. 3).

**Table 4:** Comparison of number of deaths due to bleeding avoided by tranexamic acid treatment

	Patients treated N (%) *N* = 104,862	Deaths avoided N (95% CI)	Deaths avoided per 10,000 patients N (95% CI)	Number needed to treat to avoid one death
Based on predicted probabilities				
Current strategy^a^	9915 (11)	55 (54–57)	5 (5–5)	180
All prehospital	79,430 (76)	224 (220–228)	21 (21–22)	355
All in hospital	63,246 (60)	146 (144–149)	14 (14–14)	430
Based on observed probabilities (sensitivity analysis)^b^				
Current strategy^a^	9915 (11)	168 (157–178)	16 (15–17)	59
All prehospital	79,430 (76)	323 (305–341)	31 (29–33)	244
All in hospital	63,246 (60)	240 (226–253)	22 (21–24)	273

*NNT* Number Needed to Treat

^a^Current strategy observed in the UK-TARN data based on clinical judgment and current guidelines in UK

^b^Sensitivity analysis based on observed deaths due to bleeding

**Table 5:** Number of deaths due to bleeding avoided and number needed to treat with pre-hospital treatment within 3 h of injury according to BATT score threshold as treatment criteria

BATT Score Threshold for TXA treatment	Total patients included in TARN N (%)	Number of patients considered for treatment^a^ N (%)	Number of deaths avoided by BATT score threshold	Standardized number of deaths avoided per 10,000	Number needed to treat^b^	Additional NNT^c^ for change of one point of BATT score
≥ 14	586 (< 1)	534 (< 1)	37	4.7	14	–
≥ 13	737 (< 1)	671 (< 1)	42	5.3	16	27
≥ 12	960 (1)	883 (1)	47	5.9	19	42
≥ 11	1266 (1)	1150 (1)	53	6.7	22	45
≥ 10	1727 (2)	1557 (2)	59	7.4	27	23
≥ 9	2533 (2)	2272 (2)	68	8.6	34	79
≥ 8	3859 (4)	3420 (3)	80	10.1	43	128
≥ 7	6879 (7)	5898 (6)	97	12.2	61	146
≥ 6	10,071 (10)	8584 (8)	109	13.7	78	224
≥ 5	16,032 (15)	13,335 (13)	124	15.6	108	317
≥ 4	22,946 (22)	18,769 (18)	136	17.1	138	452
≥ 3	33,483 (32)	27,062 (26)	152	19.1	179	518
≥ 2	80,071 (76)	61,598 (59)	210	26.4	293	595
≥ 1	89,948 (86)	68,452 (65)	216	27.2	316	1142
≥ 0	104,862 (100)	79,430 (76)	224	28.2	354	1372

*TXA* Tranexamic acid, *NNT* Number needed to treat

^a^Number of trauma patients within 3 h of injury and the arrival of the first ambulance. Proportions are based on all patients included in the TARN registry with ISS ≥ 9

^b^Standardized number of deaths avoided per 10,000 trauma patients within 3 h included in the TARN registry with an ISS ≥9

^c^Additional trauma patients needed to treat for each death avoided compared to the BATT score threshold above

## Discussion

### Main findings

In 2017 and 2018, only 9% of trauma patients in England and Wales received TXA and only 3% received it within an hour of injury. Pre-hospital treatment of trauma patients with a BATT score of 2 or more would substantially increase the number of premature deaths that could be avoided with TXA.

### Strengths and limitations

Our study has important strengths. Our prognostic score was derived using multivariable methods within a large international prospective cohort study with minimal missing data. We then validated the score in a second large cohort that was not used to derive the score [19]. We validated the BATT score in data from a large national trauma registry which includes trauma patients with a wide range of bleeding severity thus providing a heterogenous case-mix that allows accurate assessment of discrimination [20]. The score is based on variables recorded by paramedics at the scene of the injury when the decision to treat with TXA must be made. The large number of patients in this study increases the precision of the results. There were few missing values for predictor variables and no missing outcome data. The outcome was well defined and recorded at fixed time point. These strengths help to ensure the validity of the results.

Our study also has limitations. Measurement error of predictor variables could affect discrimination and calibration. Random error could arise for all predictors (blood pressure, heart rate, Glasgow Coma scale, Respiratory rate) and lead to reduce discrimination and calibration. Systematic errors arising from the use of monitoring devices is more likely to affect calibration [21]. Because the outcome ‘death due to bleeding’ was not available in TARN database, we used early death as a proxy for death due to bleeding [22]. However, any outcome misclassification would be expected to decrease the C-statistic and reduce model performance [23] and since the C-statistic was high and model performance was excellent, misclassification is unlikely to be an important weakness. Because time from injury to ambulance arrival and hospital admission was missing for nearly half of the patients, we imputed these data. Misclassification of time to treatment could affect our estimate of the net benefit [24]. The estimates of deaths avoided are unlikely to be generalizable since they depend on the risk of death, which may vary in different settings. To model the number of deaths avoided, we used treatment effect estimates from randomised trials and so the estimates should be unconfounded. However, confounders in this observational study might affect our estimates of the absolute number of deaths avoided and so this must be considered with caution. Because we used the same method to estimate the impact of each strategy, it is unlikely that the comparison between different strategies was adversely affected by potential confounders. Furthermore, we are reassured by the result of the STAAMP trial assessing TXA in trauma patient in the prehospital setting [25]. The magnitude of the treatment effect observed in this trial is similar to that observed in the CRASH-2 trial although the estimate was more imprecise.

### Relation to other studies

To the best of our knowledge, ours is the only score that predicts traumatic death due to bleeding. Existing haemorrhage scores predict massive transfusion which is an imperfect surrogate of death due to bleeding and vulnerable to survival bias (i.e. TASH score, ABC score) [26, 27].

### Clinical implications

Clinical guidelines recommend TXA treatment for patients with or at risk of significant bleeding and that treatment is given as soon as possible [3]. Due to the lack of clear treatment criteria, many trauma patients are not receiving TXA or else receive it too late. A study on paramedic perceptions concerning tranexamic acid use in bleeding in trauma patients showed that lack of self-confidence, uncertainty about haemorrhage risk and the need to give TXA by slow intravenous injection (over 10 min) were the main barriers to TXA administration [28]. Our data suggest that using a BATT score threshold of 2 or more would improve outcomes with a fourfold increase in bleeding deaths prevented by TXA. This clear criterion could improve prehospital administration of TXA by paramedics. Although the use of this threshold would increase the number of patients treated, TXA is safe and inexpensive and is likely to be highly cost-effective [29, 30]. Randomised trials of TXA in trauma and surgery have included over 50,000 patients and no increase in vascular occlusive events has been found [4, 17, 31–33]. Recent trials in prehospital trauma did not find any increase in vascular occlusive events associated with TXA and provide evidence for applicability of TXA treatment in the prehospital setting [25, 34].

Recent research has found that TXA is well tolerated and rapidly absorbed after intramuscular injection reaching therapeutic concentrations within 15 min in bleeding trauma patients [35]. Further research is needed to assess the cost-effectiveness of different treatment thresholds and whether use of the BATT score and intramuscular TXA administration by paramedics increases the pre-hospital administration of TXA to patients at risk of bleeding from trauma. Prospective validation of the BATT score would certainly increase its value for clinical use.

## Conclusion

The BATT score is a validated tool, easy to perform at the scene of injury to identify trauma patients at risk of death from bleeding. A score of 2 or more would be an appropriate threshold for pre-hospital tranexamic acid treatment.

## Supplementary Information

**Additional file 1: Supplementary file 1.** Abbreviated Injury Scale diagnosis associated with haemorrhage. **Supplementary file 2.** Formula for the Brier Score and Scaled Brier Score. **Supplementary file 3.** Methods to model tranexamic acid treatment effect and death due to bleeding avoided. **Supplementary file 4.** Receiving Operator Curve for external validation of the BATT score. **Supplementary Figure 5.** Sensitivity and specificity according to BATT score for death due to bleeding. **Supplementary file 6.** Calibration curve for external validation of the BATT score.

## Data Availability

Data are available on reasonable request and with agreement from TARN.
