# Supplementary material for: Validation of the BATT score for prehospital risk stratification of traumatic haemorrhagic death: usefulness for tranexamic acid treatment criteria
Source: Scand J Trauma Resusc Emerg Med. 2021 Jan 6;29:6. doi: 10.1186/s13049-020-00827-5 (PMC7789642; doi:10.1186/s13049-020-00827-5)

Additional file 1

Supplementary file 1. Abbreviated Injury Scale diagnosis associated with haemorrhage

- Blood loss > 20%.

- Aorta [OR] Vena Cava [OR]carotid [OR]femoral [OR]Major arteries [OR]veins AND laceration.

- Spleen [OR]liver [OR] Kidney [OR] Myocardium [AND] major laceration.

- Major haemothorax.

- Retroperitoneum haemorrhage.

Supplementary file 2. Formula for the Brier Score and Scaled Brier Score

Brier Score= $\frac{1}{N}\sum_{i=1}^{n}(Y-p)^2$

Which Y is the observed outcome and p the prediction of the model.

Brier Score$_{max}$= $P \times (1-P)^2 + (1-P) \times P^2$

Which P is the mean of the prediction p.

Scaled Brier score= $\frac{1-Brier}{Brier\ max}$

Scaled Brier score ranges from 0% to 100%

Supplementary file 3. Methods to model tranexamic acid treatment effect and death due to bleeding avoided.

First method

a) We estimated the baseline probabilities of death due to bleeding in the TARN population (P1).

P1= [0.5344157 - 0.5726779 + (0.0604783 * age) - (0.0013908 * age2) + (0.000012 * age3) + (0.0234826 * isbp) - (0.0005366 * isbp2) + (0.00000158 * isbp3) - (0.6336347 * igcs) + (0.0738416 * igcs2) - (0.0029216 * igcs3) - (0.0085677 * ihr) + (0.0001027 * ihr2) - (0.1709854 * irr) + (0.0059866 * irr2) - (0.000054 * irr3) + (0.3056116 * penetrating)] * 0.82

P1 (Baseline probabilities of death due to bleeding); ISBP (initial systolic blood pressure); IGCS (initial Glasgow coma scale); IHR (initial heart rate); IRR (initial respiratory rate); Penetrating injury.

b) We used previous studies exploring treatment effect by time and baseline risk (TE).

TE= OR txa/time * OR txa/baseline risk

TE (treatment effect); OR (Odds ratio)

OR txa/time is function of delay from Accident to Ambulance Arrival (Prehospital treatment) or Delay from Accident to Hospital Arrival (In-hospital treatment). (REF Lancet Gayet)

0.70235307 if delay=0 min
0.70698462 if delay=5 min
0.71164609 if delay ==10 min
0.71633767 if delay ==15 min
0.72105956 if delay ==20 min
0.72581194 if delay ==25 min
0.73059501 if delay ==30 min
0.73540897 if delay ==35 min
0.740254 if delay ==40 min
0.7451303 if delay ==45 min
0.75003808 if delay ==50 min
0.75497752 if delay ==55 min
0.75994883 if delay ==60 min
0.76495222 if delay ==65 min
0.76998788 if delay ==70 min
0.77505601 if delay ==75 min
0.78015683 if delay ==80 min
0.78529054 if delay ==85 min
0.79045734 if delay ==90 min
0.79565744 if delay ==95 min
0.80089106 if delay ==100 min
0.80615841 if delay ==105 min
0.81145969 if delay ==110 min
0.81679513 if delay ==115 min
0.82216493 if delay ==120 min
0.82756932 if delay ==125 min
0.83300851 if delay ==130 min
0.83848272 if delay ==135 min
0.84399218 if delay ==140 min
0.84953709 if delay ==145 min
0.8551177 if delay ==150 min
0.86073421 if delay ==155 min
0.86638687 if delay ==160 min
0.87207589 if delay ==165 min
0.87780151 if delay ==170 min
0.88356395 if delay ==175 min
0.88936344 if delay ==180 min

OR txa/baseline risk is constant=1 (Ref BJA)

c) We estimated Post-Treatment probabilities of death due to bleeding (P2)

P2=P1 * TE

d) We estimated the number of death due to bleeding avoided by tranexamic acid.

Number of death avoided= $\sum P1 - \sum P2$

e) Net benefit

Net benefit= Number of death avoided – Number of death due to side effect

We considered tranexamic acid treatment within 3 hours from injury. In this time interval, we did not find any randomized control trial reporting death due to side effect or any increase of non-fatal vascular occlusive event.

Net Benefit = Number of death avoided

Sensitivity analysis (Second method)

a) We estimated the baseline probabilities of death due to bleeding in the TARN population ($P1_{obs}$).

We divided death due to bleeding by treatment effect for patient treated by tranexamic acid to estimate baseline probabilities.

$$P1_{obs} = (Death_{obs})_{if\ TXA=0} + \left(\frac{Death_{obs}}{TE}\right)_{if\ TXA==1}$$

$Death_{obs}$=Early death with evidence of haemorrhage

b) We estimated Post-Treatment probabilities of death due to bleeding (P2)

$$P2 = P1_{obs} * TE$$

c) We estimated the number of death due to bleeding avoided by tranexamic acid.

$$\text{Number of death avoided} = \sum P1 - \sum P2$$

d) Net benefit

Net benefit= Number of death avoided – Number of death due to side effect

We considered tranexamic acid treatment within 3 hours from injury. In this time interval, we did not find any randomized control trial reporting death due to side effect or any increase of non-fatal vascular occlusive event.

Net Benefit = Number of deaths avoided

Supplementary file 4. Receiving Operator Curve for external validation of the BATT score.

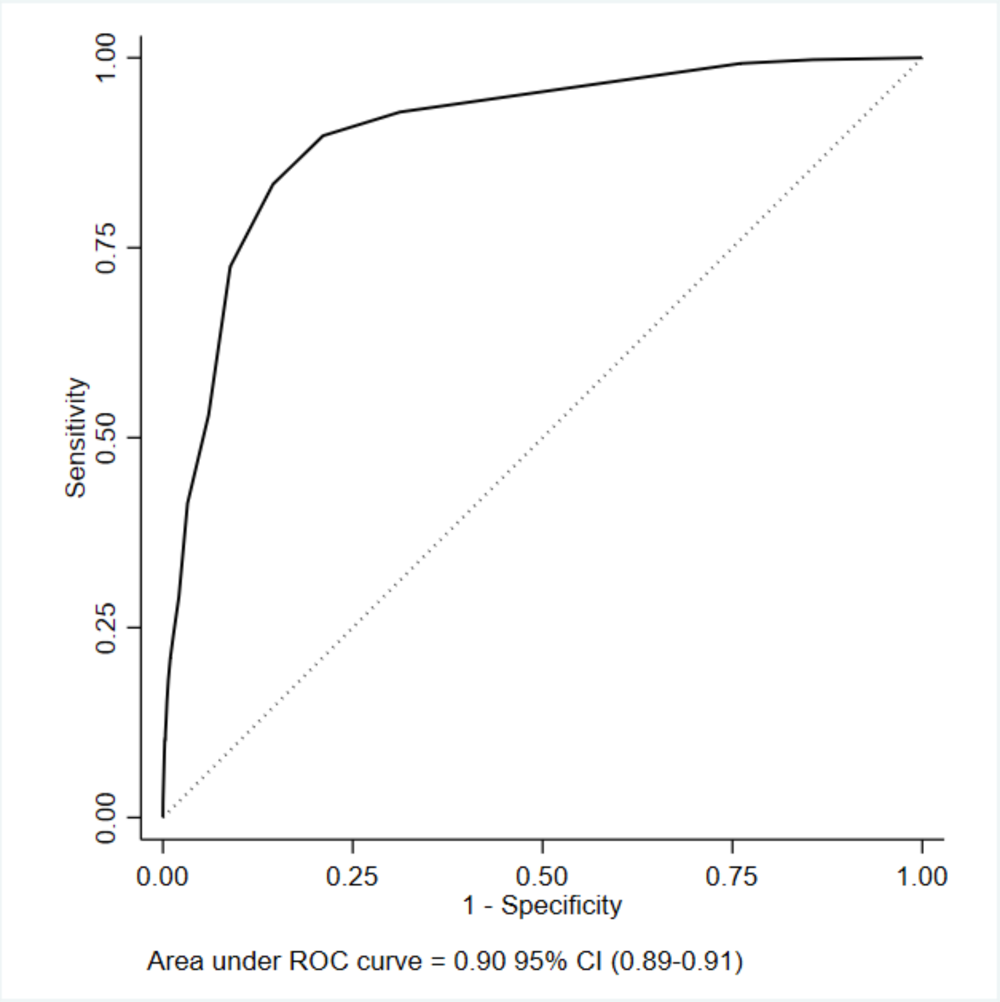

Supplementary figure 5. Sensitivity and specificity according to BATT score for death due to bleeding.

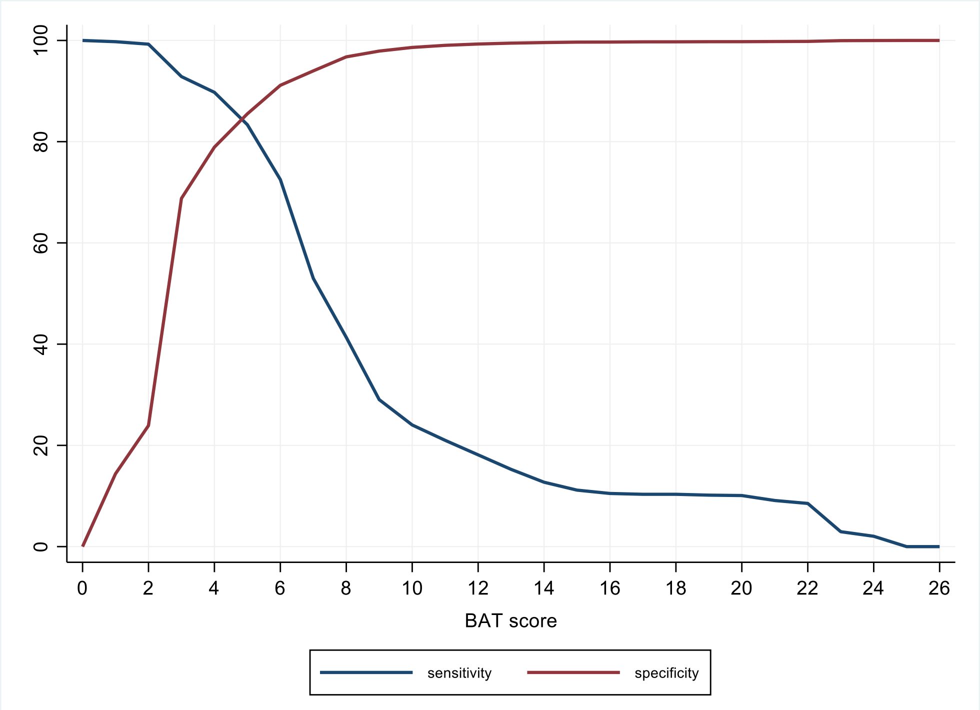

Threshold	Sensitivity (%)	Specificity (%)	Likelihood ratio +	Likelihood ratio -
0	100	0	1	-
≥ 1	100	14	1.17	0.017
≥ 2	99	24	1.31	0.031
≥ 3	93	69	2.98	0.104
≥ 4	90	79	4.26	0.130
≥ 6	73	91	8.18	0.302
≥ 8	41	97	12.77	0.606
≥ 10	24	99	17.37	0.770
≥ 12	18	99	25.42	0.825

Supplementary file 6. Calibration curve for external validation of the BATT score.

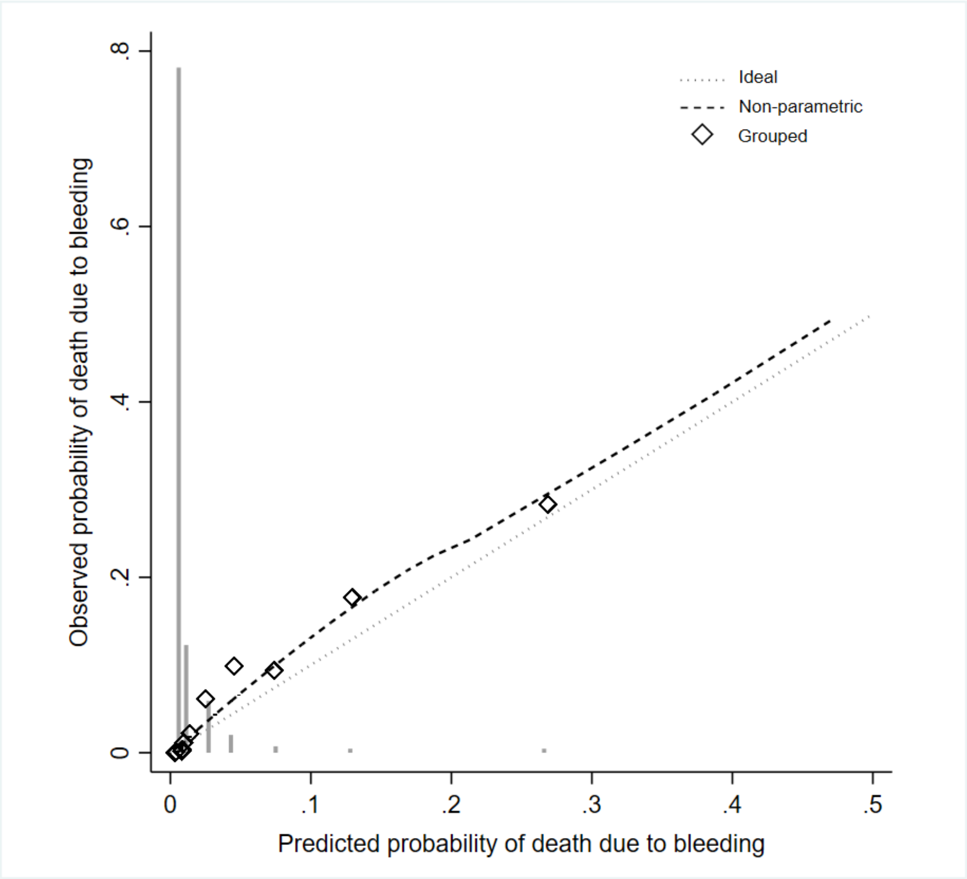

Supplement: Supplementary file 1 — Additional file 1: Supplementary file 1. Abbreviated Injury Scale diagnosis associated with haemorrhage. Supplementary file 2. Formula for the Brier Score and Scaled Brier Score. Supplementary file 3. Methods to model tranexamic acid treatment effect and death due to bleeding avoided. Supplementary file 4. Receiving Operator Curve for external validation of the BATT score. Supplementary Figure 5. Sensitivity and specificity according to BATT score for death due to bleeding. Supplementary file 6. Calibration curve for external validation of the BATT score. [file 13049_2020_827_MOESM1_ESM.pdf]
